# Genetic and histologic spatiotemporal evolution of recurrent, multifocal, multicentric and metastatic glioblastoma

**DOI:** 10.1186/s40478-020-0889-x

**Published:** 2020-02-03

**Authors:** Maria-Magdalena Georgescu, Adriana Olar

**Affiliations:** 1 NeuroMarkers PLLC, Houston, TX 77025 USA; 2 Department of Pathology and Neurosurgery, Medical University of South Carolina and Hollings Cancer Center, Charleston, SC 29425 USA

**Keywords:** Metastatic glioblastoma, Next generation sequencing, Recurrent, Multifocal, Multicentric, Gliosarcoma, Epithelioid, Invasion, PTEN

## Abstract

Glioblastoma is the most frequent and aggressive primary brain tumor, characterized by extensive brain invasion and rarely, systemic metastases. The pathogenesis of metastatic glioblastoma is largely unknown. We present the first integrated clinical/histologic/genetic analysis of 5 distinct brain and lung foci from a unique case of recurrent, multifocal, multicentric and metastatic glioblastoma. The initial right frontotemporal gliosarcoma received standard surgical/chemoradiation therapy and recurred 1.5 years later, co-occurring with three additional masses localized to the ipsilateral temporal lobe, cerebellum and lung. Synchronous metastatic lung carcinoma was suspected in this long-term smoker patient with family history of cancer. However, glioblastoma was confirmed in all tumors, although with different morphologic patterns, including ependymomatous and epithelioid. Genomic profiling revealed a germline *FANCD2* variant of unknown significance, and a 4-gene somatic mutation signature shared by all tumors, consisting of *TERT* promoter and *PTEN, RB1* and *TP53* tumor suppressor mutations. Additional *GRIN2A* and *ATM* heterozygous mutations were selected in the cerebellar and lung foci, but were variably present in the supratentorial foci, indicating reduced post-therapeutic genetic evolution in brain foci despite morphologic variability. Significant genetic drift characterized the lung metastasis, likely explaining the known resistance of circulating glioblastoma cells to systemic seeding. *MET* overexpression was detected in the initial gliosarcoma and lung metastasis, possibly contributing to invasiveness. This comprehensive analysis sheds light on the temporospatial evolution of glioblastoma and underscores the importance of genetic testing for diagnosis and personalized therapy.

## Introduction

Glioblastoma is the most frequent and aggressive primary brain tumor in adults, with an incidence of 3–4 cases per 100,000 population, and a median survival of approximately 1 year [18]. Although glioblastoma is one of the most heterogeneous human neoplasms, only three morphologic variants are recognized in the 2016 World Health Organization (WHO) Classification of Tumors of the Central Nervous System (CNS): gliosarcoma (2% of glioblastomas), giant cell glioblastoma (less than 1% of glioblastomas), and epitheliod glioblastoma (rare, incidence not known) [18]. Genetically, gliosarcoma differs from primary/IDH wild-type glioblastoma by a slightly higher *PTEN* mutation rate and infrequent *EGFR* alterations [29], whereas epithelioid glioblastoma may harbor *BRAF* V600E mutation in approximately half of the cases [15]. Of these, gliosarcoma has been reported to have a higher than expected rate of systemic metastasis [2, 19].

The dismal prognosis in glioblastoma is due to tumor heterogeneity but also to the invasiveness of the tumor cells within normal brain, which leads to resistance to the current surgical, radiologic and chemotherapeutic approaches. Glioblastoma is suspected radiologically based on the magnetic resonance imaging (MRI) appearance of a rim/ring-enhancing mass on T1-weighted (W) post-contrast studies that corresponds to a central area of necrosis surrounded by viable tumor with disrupted blood-brain barrier. From this tumor core, the neoplastic cells invade normal brain, inducing surrounding T2W-fluid attenuated inversion recovery (FLAIR) hyperintensity without corresponding T1W post-contrast enhancement. Occasionally, secondary hyperproliferative and/or necrotic foci showing contrast enhancement develop, resulting in multifocal or multicentric glioblastoma, depending on the presence or absence of the T2-FLAIR hyperintensity connecting the contrast-enhancing foci, respectively. Very rarely, glioblastoma may become metastatic to extra-neural sites, with approximately 300 cases reported in the literature [1, 25, 31]. Even if this number accounts for approximately 1% of glioblastoma cases, the pathogenesis and management of metastatic glioblastoma are largely unknown and there is no comprehensive genomic characterization of these cases. In this study, we performed an integrated clinical, histologic and genomic analysis of 5 distinct surgical foci from a patient receiving standard treatment before developing recurrent multifocal, multicentric and metastatic glioblastoma. This analysis revealed high morphological variability in the absence of a high tumor mutation burden (TMB) during the intraneural spatiotemporal evolution of glioblastoma. Importantly, it showed a striking accumulation of mutations in the lung metastasis, leading to significantly increased TMB and strong activation of the PI3K/PTEN/AKT and p53 pathways, with critical pathogenic and therapeutic implications.

## Materials and methods

### Histology and immunohistochemistry (IHC)

Formalin-fixed paraffin-embedded (FFPE) sections from brain tumor resection and lung needle biopsy specimens were stained with hematoxylin-eosin (H&E). Images were acquired with a Nikon Eclipse Ci microscope equipped with a Nikon Digital Sight DS-Fi2 camera (Nikon Instruments Inc., Melville, NY), as previously described [10]. IHC was performed with clinically validated antibodies on a Ventana Benchmark Ultra platform (Roche/Ventana Medical Systems Inc., Tucson, AZ) [10]. The primary antibodies were: glial fibrillary acidic protein (GFAP) (EP672Y), Olig-2 (387 M-15) (Ventana/Cell Marque, Rocklin, CA), p53 (DO-7), Ki-67 antibody (30–9) (Roche/Ventana Medical Systems Inc.), IDH1-R132H (DIA-H09, Dianova, Hamburg, Germany) and NHERF1/EBP50 (Thermo/Fisher, Waltham, MA). The automated Ki-67 proliferation index was performed by using the Nikon NIS Elements 4.51.00 program set up with an object-count algorithm for recognition of differentially labeled nuclei, as previously described [32].

### Next generation sequencing (NGS) and copy number (CN) analysis

FFPE tumor tissue from a representative block with or without microdissection was used for nucleic acids extraction for NGS or microarray-based CN analysis, respectively. Saliva was collected for normal matched control. All samples were sequenced at Tempus Labs (Chicago, IL), by using a 596-gene panel (Additional file 1: Table S1), as previously described [8]. The limit of detection of the assay is 5% variant allele fraction (VAF), with sensitivity of 99.1% for single nucleotide variants. Variant analysis and interpretation was performed by using Tempus proprietary software, ClinVar (National Institutes of Health, Bethesda, MD), Catalogue of Somatic Mutations in Cancer (COSMIC) and VarSome [16]. The final somatic VAF was adjusted depending on the tumor cell content of the sample. Loss of heterozygosity (LOH) was called for clonal mutations with gene locus CN loss. TMB represents the number of single nucleotide protein-altering mutations per million base pairs. Graphs were plotted by using GraphPad Prism (Version 8.3.1, GraphPad Software, La Jolla, CA). Microarray-based chromosome CN analysis was performed by using the IScan® System with the CytoSNP-850 K v1.1 BeadChip (Illumina, San Diego, CA), and GenomeStudio (Illumina) and Nexus, (Version 9.0, BioDiscovery, Inc., El Segundo, CA) softwares, as previously described [8].

### Three-dimensional (3D) modeling

The human FANCD2 wild-type and mutant residues were mapped in the 3D structure of the mouse FANCD2-FANCI complex (Protein Data Base accession number: 3s4w [14]). The models were generated by using PyMol Molecular Graphics System (Version 2.3.0, Schrodinger, LLC), as previously described [11, 21].

## Results

### Clinical approach to diagnosis and treatment in a glioblastoma patient with multiple neural and extra-neural masses

**Fig. 1:**
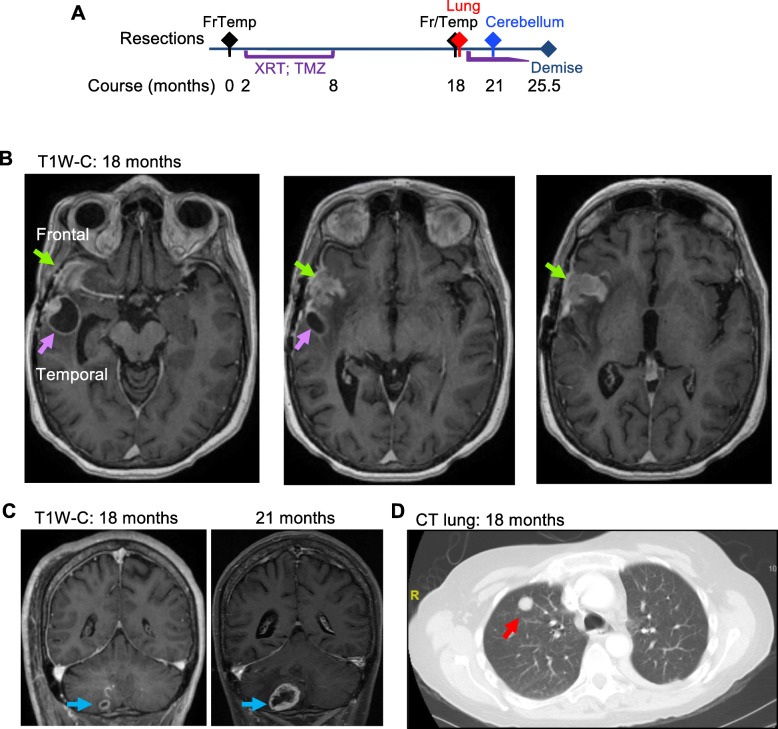
Clinical case presentation. **a** Time course of the patient’s clinical progression: the initial right frontotemporal (FrTemp) mass was surgically removed and the patient received standard radiotherapy (XRT) and temozolomide (TMZ). At 18 months postresection, 4 new ipsilateral masses occurred: frontal (Fr), temporal (Temp), as well as in the lung and cerebellum near midline, and underwent resections, followed by chemotherapy. **b.** MRI at the time of recurrence, 18 months following the 1st craniotomy for the initial mass resection. Serial axial T1W post-contrast (T1W-C) images show a multifocal mass comprising a frontal mass (Frontal, green arrows) at the site of the previous resection, and a temporal mass (Temporal, purple arrows). **c** Coronal T1W post-contrast (T1W-C) images showing rapid growth of a cerebellar mass (blue arrows) between the 2nd and the 3rd craniotomies. **d** Chest CT with contrast showing a solid mass in the right lung (red arrow)

A 49-year-old White female, without significant past medical history, presented for headaches and left-sided weakness for 6 weeks. Her social history was significant for smoking 1 pack/day for 20 years. Her family history included mother deceased of lung cancer, and sister deceased of breast cancer, diagnosed at 27 years of age. Computed tomography (CT) imaging revealed a 4.6 × 4.5 × 3.8 cm right frontotemporal, intra-axial, heterogeneous mass with associated edema, 1 cm right-to-left subfalcine shift, and uncal herniation. After maximal surgical resection, a histologic diagnosis of glioblastoma, WHO grade IV, was rendered. The patient’s left-sided weakness improved considerably following surgery and she complained only of mild headache subsequently. Two months post-resection, she received concurrent temozolomide (75 mg/m^2^ per os daily) and fractionated radiotherapy (total 60 Gy) for 6 weeks, followed by 6 cycles of temozolomide maintenance for the next 6 months (150 mg/m2 per os daily for 5 days; repeat cycle every 4 weeks) (Fig. 1a). At approximately 18 months after the initial diagnosis, the patient developed local recurrence noted on MRI that appeared to have two components, a homogenously enhancing mass at the previous resection site (Fig. 1b, green arrows; frontal mass) and an adjacent ring enhancing cystic mass (Fig. 1b, purple arrows; temporal mass). A small, right cerebellar, rim-enhancing mass, without obvious connection to the ventricular system or leptomeninges, as sites of cerebrospinal fluid (CSF) metastatic seeding, was also noted at that time (Fig. 1c). Concomitantly, chest CT identified an incidental, well-defined, solid, nodular lesion in the right upper lobe, measuring 1.4 cm in diameter, suspicious for malignancy (Fig. 1d). A clinical diagnosis of glioblastoma recurrence and the possibility of a concomitant primary lung malignancy metastatic to the brain was formulated in this patient with smoking history. To confirm this possibility and direct therapy, the patient underwent right frontotemporal craniotomy with partial mass resection of both supratentorial tumor components (Additional file 1: Figure S1A), followed by lung mass needle biopsy. Due to rapid growth of the cerebellar tumor (Fig. 1c and Additional file 1: Figure S1B), a third craniotomy with complete cerebellar mass resection was undertaken at 21 months after the first craniotomy, and 3 months after the second one. After recovery, the patient resumed temozolomide chemotherapy. She succumbed 4.5 months after the last craniotomy, with a total survival of 25.5 months after the initial diagnosis.

### Histopathologic examination reveals metastatic, multifocal and multicentric glioblastoma

**Fig. 2:**
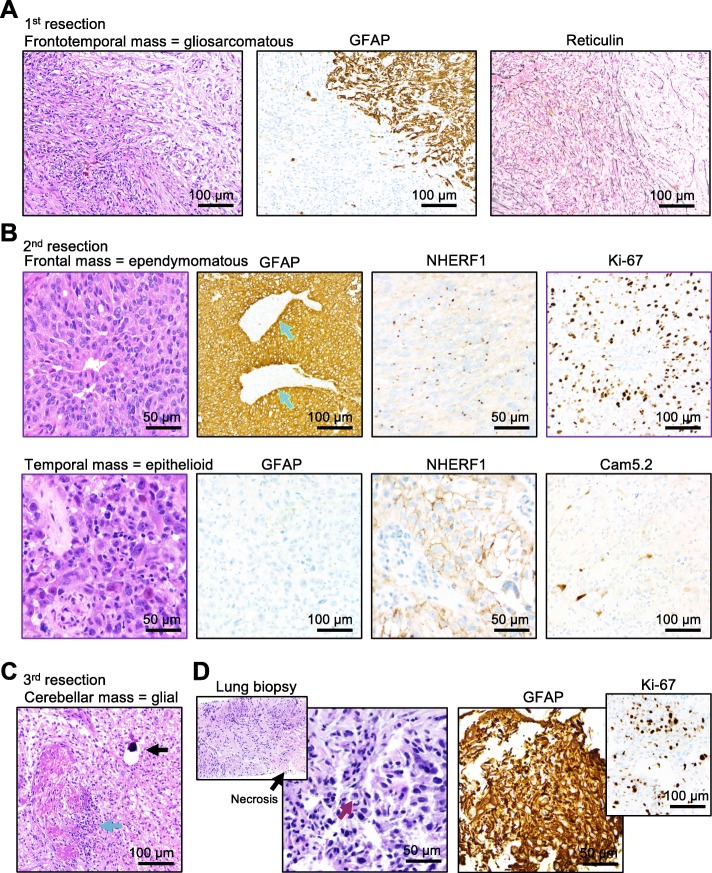
Morphologic variability of glioblastoma diagnosed in all five tumors. **a** H&E, GFAP immunolabeling and reticulin special staining show the biphasic pattern of gliosarcoma in the initial frontotemporal mass: the glial component (right upper corner) expresses GFAP and lacks pericellular reticulin deposition, whereas the sarcomatous component (left lower corner) shows opposite phenotype. **b** Ependymomatous and epithelioid morphology of the frontal and temporal recurrent tumors from the 2nd resection, respectively. Note opposite GFAP labeling patterns with perivascular tumor cell arrangement in the frontal mass (vessels indicated with green arrows), NHERF1 dot-like microlumen labeling in the frontal mass and membranous staining in the temporal mass, high Ki-67 proliferation in the frontal mass, and very focal Cam5.2 cytokeratin staining in the temporal mass. **c** Resection H&E of the cerebellar mass shows a homogenous glial component, calcifications (black arrow) and damaged vessels obstructed by fibrin thrombi and with invasion of neoplastic cells (blue arrow). **d** The lung biopsy H&E shows fibrillary areas (inset) with necrosis (black arrow), and pleomorphic areas with brisk mitotic activity (red arrow) and high Ki-67 proliferation index. GFAP diffusely labels the entire tumor

Diagnostic material was obtained from all 5 tumors and was unexpectedly consistent with several histologic variants of glioblastoma, IDH wild-type, WHO grade IV (Fig. 2 and Additional file 1: Table S2). The majority of the viable tumor from the first resection exhibited the biphasic morphological and staining pattern of gliosarcoma, with mutually exclusive GFAP-positive glial and reticulin-positive sarcomatous areas (Fig. 2a, Frontotemporal mass). Extensive necrosis, microvascular proliferation, vessels with fibrin thrombi and mitotic figures were present. Unexpectedly, the recurrence at the site of the previous resection (Fig. 2b, Frontal mass, see also Fig. 1b) had a predominant leptomeningeal component with ependymomatous morphology, including pseudorosetting and canal-like spaces, strong and diffuse GFAP staining and formation of microlumens labeled by NHERF1 [12]. The surrounding brain parenchyma was massively infiltrated by neoplastic cells with focal myxoid extracellular matrix formation. The adjacent temporal rim-enhancing tumor (Fig. 2b, Temporal mass, see also Fig. 1b) had a striking epithelioid morphology, with large epithelioid cells containing nuclei with prominent nucleoli, absence of GFAP labeling, focal plasma membrane NHERF1 labeling, as in papillary lung adenocarcinoma [9], and very focal low molecular weight cytokeratin CAM5.2 and Olig2 expression. This predominant appearance was consistent with the epithelioid morphologic variant of glioblastoma [18]. The morphology of the cerebellar mass resembled the glial component of the initial gliosarcoma (Fig. 2c and Additional file 1: Table S2). Calcifications were seen in this specimen, suggesting a protracted course, most likely prior to the rapid growth observed after the 2nd temporal craniotomy. Vessels with fibrin thrombi invaded by tumor cells were numerous (Fig. 2c, blue arrow). The lung biopsy showed a heterogeneous tumor with diffuse GFAP expression, that exhibited areas of pleomorphic cells embedded in a myxoid extracellular matrix showing brisk mitotic activity, and areas of fibroblastic-like cells with lower mitotic activity (Fig. 2d). These findings uncovered an unexpected spatiotemporal morphological variation in the different foci of the same malignancy.

### Genetic analysis detects a common signature in all foci and significant mutation accumulation in the extra-neural lung metastasis

**Fig. 3:**
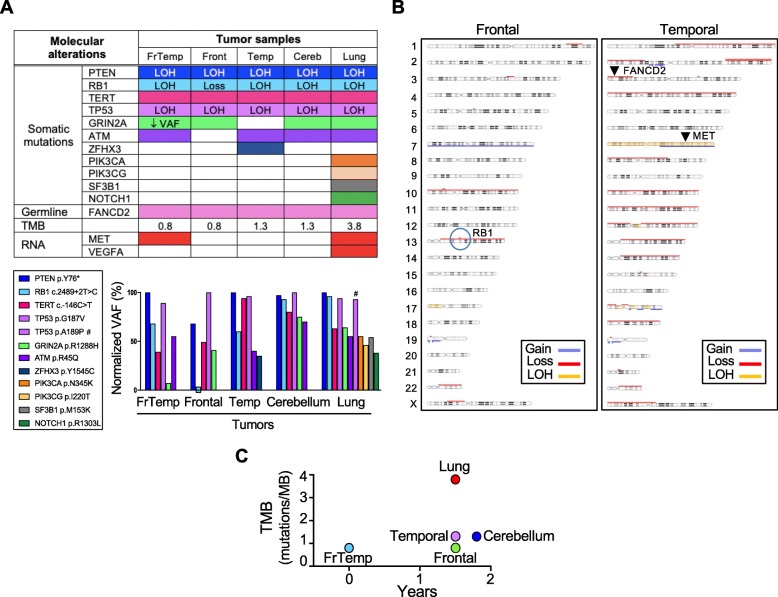
Genomic profiling. **a** Summary table of genomic and transcriptomic analysis, and bar graph of somatic mutation composition and VAF. Color-coded shading indicates mutant variants, whereas white rectangles correspond to wild-type variants. RNA overexpression is shown by red rectangles. The VAF of individual mutations was normalized to the highest VAF in a given sample, that was arbitrarily set to 100%, due to LOH (see Material and methods). FrTemp, frontotemporal (initial mass); Front, frontal; Temp, temporal; Cereb, cerebellum; Loss, homozygous CN loss; #, additional *TP53* mutation in the lung specimen. **b** Chromosomal CN array from the adjacent frontal and temporal recurrences showing common chromosomal alterations but also divergence of the temporal tumor by extensive chromosome losses. Both tumors have loss of chromosome 10, and the frontal mass has gain of chromosome 7, whereas the temporal mass has a more complex CN gain and LOH of chromosome 7. The *RB1* locus with homozygous CN loss is encircled, and the loci for *FANCD2* and *MET* are indicated by arrowheads. **c** TMB representation showing significant mutation accumulation in the lung mass

To confirm the common origin of the five tumors and track their genetic spatiotemporal evolution, NGS was performed on all five tumor samples, as well as matched normal saliva (Fig. 3a). A common 4-gene somatic mutation signature was identified in all five tumors, confirming their origin from the initial gliosarcoma. A *PTEN* p.Y76* truncating mutation with LOH was predicted to completely eliminate PTEN expression and therefore activate the PI3K/AKT pathway [7]. A pathogenic, loss of function *RB1* splice variant (c.2489 + 2 T > C) with LOH was present in all tumors, except for the frontal recurrence, where a homozygous CN loss spanned the *RB1* locus on chromosome 13q14.2 (Fig. 3b, encircled). *TERT* promoter mutation, the most common genetic alteration in glioblastoma [18], was found at position c.-146C > T. A missense mutation in *TP53* gene with LOH, resulting in a splice region variant (c.560G > T; p.G187 V) was classified as pathogenic. This variant has been described as somatic mutation in rectal cancer [28], and germline mutation in cancer predisposing syndromes, including Li-Fraumeni (ClinVar, 3 submitters).

Other mutations were restricted to only few of the tumors, or were focal, in just one tumor (Fig. 3a). *GRIN2A* (Glutamate Receptor, Ionotropic, N-Methyl D-Aspartate 2A) encodes the GluN2A receptor subunit that has been shown to act as a tumor suppressor, with mutant forms acting in a dominant negative fashion on the wild-type receptor [27]. The p.R1288H somatic mutation in *GRIN2A* has been extensively reported in various solid cancers, including melanoma, lung and colorectal cancer [34]. This mutation was present at low VAF in the initial frontotemporal tumor, and was selected at heterozygous VAF in three other tumors (Fig. 3a, bar graph), suggesting genetic variability in the initial tumor with subsequent clonal selection. An *ATM* p.R45Q missense mutation was found in 4 samples, not being detected in the frontal recurrence. Missense mutations at this residue were identified in the germline of patients with cancer predisposing syndromes, including ataxia-teleangiectasia (AT) syndrome (ClinVar, 8 submitters). Its lack in the frontal recurrence in the absence of chromosomal aberrations at the *ATM* 11q22.3 locus (Fig. 3b and Additional file 1: Table S3) suggests that this tumor evolved from a subpopulation of the initial frontotemporal tumor distinct from the one that gave rise to the rest of the foci. A somatic missense mutation p.Y1545C of unknown significance in *ZFHX3* (Zinc Finger Homeobox 3), a transcription factor regulating neuronal differentiation with tumor suppressor role, was found only in the temporal tumor.

The lung metastatic focus acquired a multitude of distinct somatic missense mutations, as well as a significantly increased TMB, as compared to the 4 brain foci (Fig. 3a and c). The presence of an additional pathogenic mutation in *TP53*, p.A189P, co-existing on the same allele just two codons downstream from the common p.G187 V mutation (Fig. 3a, bar graph), of two mutations in the related PI3K genes, *PIK3CA* p.N345K and *PIK3CG* p.I220T, of a p.M153K mutation of unknown significance in *SF3B1* splicing factor gene, known to be mutated in several malignancies, and a likely pathogenic mutation in *NOTCH1,* p.R1303L, previously described in squamous cell carcinoma [33], indicated much higher variability in this focus compared to the relatively stable brain foci.

A *FANCD2* p.I273V missense variant of unknown significance was the only germline mutation detected by using the 596-gene panel in this patient with family history of cancer (Fig. 3a). This genetic germline alteration has been previously found in Fanconi Anemia (FA) (ClinVar, Invitae, one entry, affected status unknown), a genetic disease characterized by high incidence of severe bone marrow failure, cancer, and various congenital defects [23]. The residue Ile273 maps at the tip of a loop connecting an α-helix involved in the interface between FANCD2 and FANCI [14] and its change to Val appears to mildly restrict the hydrophobicity of the protein surface (Additional file 1: Figure S2). This modification is unlikely to alter the interaction between FANCD2 and FANCI, but an influence on other FANCD2 interactors cannot be excluded [3, 35].

*MGMT* promoter was not methylated. Transcriptomic analysis showed *MET* upregulation in the initial gliosarcoma and in the lung metastasis (Fig. 3a). Additional VEGFA overexpression was present in the lung metastasis but the secondary brain foci were relatively quiescent.

Although the mutational signature and TMB were relatively conserved, the CN alterations were strikingly divergent between the two adjacent supratentorial brain foci at the recurrence time (Fig. 3b and Additional file 1: Table S3). Both carried loss of chromosome 10 and gain or partial gain of chromosome 7, a cytogenetic abnormality relatively specific for glioblastoma [18]. The temporal tumor with epithelioid morphology had multiple chromosomal losses, including at the *FANCD2* locus (Fig. 3b), indicating chromosomal instability.

## Discussion

**Fig. 4:**
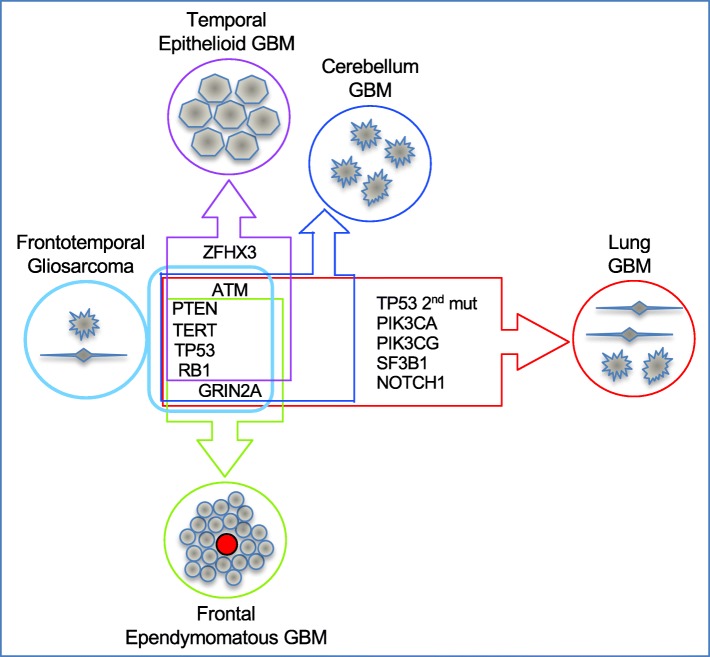
Genetic and morphologic spatiotemporal evolution model. Tumors are schematically shown as circles filled with cells of various morphologies. The initial gliosarcoma is shown in light blue color on the left, adjacent to a box representing its genetic composition of core clonal mutations on the left, and subclonal mutations on the right. The genetic drift is shown on the vertical axis for the closely related intraneural secondary/recurrent foci, and on the horizontal axis for the more divergent lung metastasis. Additional mutations are indicated in the corresponding tumor boxes, whereas the 4-gene core signature is common to all tumors. GBM, glioblastoma

The molecular characterization of glioblastoma is instrumental for an accurate diagnosis and to inform therapeutic decisions. Due to difficult accessibility, there is very limited information on the molecular spatiotemporal evolution of glioblastoma and its response to therapy, with only recent efforts to characterize matched primary and recurrent tumors [30]. We analyzed comprehensively the spatiotemporal evolution of a complex case of recurrent glioblastoma with multifocality, multicentricity and extraneural lung metastasis, and noted two opposite genetic evolution pattern in the intra-neural and extra-neural compartments (Fig. 4). The three intra-neural foci occurring post-therapy showed only mild temporospatial mutational divergence without significant TMB change, regardless of their supratentorial or infratentorial location. In contrast, the lung focus showed major genetic drift with significantly increased TMB and surprisingly, accumulation of pathogenic mutations in pathways already activated in the initial tumor, such as the PI3K/PTEN/AKT and p53. This mutation shift may explain the postulated resistance of glioblastoma circulating cells to establish systemic metastases in immunocompetent individuals [22], and we hypothesize that it is required for the adaptation of the metastatic tumor to a different microenvironment. Whereas the four core mutated genes are among the most frequently mutated genes in glioblastoma [18], the prevalence of the non-core mutated genes is low, as found in The Cancer Genome Atlas (TCGA) glioblastoma cohorts (Additional file 1: Table S4). In comparison, *GRIN2A* mutations have relatively higher prevalence in TCGA cohorts of lung adenocarcinoma, squamous cell carcinoma, or in cutaneous melanoma, and *PIK3CA* mutations, in lung adenocarcinoma and squamous cell carcinoma. Although pathogenic mutations in *PTEN* and *PIK3CA* are generally mutually exclusive in glioblastoma, they may coexist in other solid cancers, strongly activating the PI3K/PTEN/AKT pathway [6]. Similar AKT activation has been shown for invasive glioblastoma cells from secondary foci in a mouse model [20]. Interestingly, accumulation of a distinct mutation in *TP53* has been previously reported in a metastatic glioblastoma case [25], but because of the limited analysis, this was supposed to be deriving from a subclone rather than being part of an acquired major genetic shift, as we propose.

Based on the trace VAF of *GRIN2A* mutation in the initial gliosarcoma and on the absence of *ATM* mutation in the frontal recurrence, the initial tumor most likely contained few genetically distinct subpopulations that gave rise to the various subsequent tumors (Fig. 4). It is interesting that all these subpopulations contained a low TMB with just four basic driver mutations in *RB1*, *TP53*, *TERT* and *PTEN*, that disabled the cell cycle checkpoints conferring uncontrolled proliferation. Similar to the lung metastasis, it is possible that the recurrent adjacent frontal and temporal masses that differed genetically more between them than with the initial frontotemporal tumor, acquired and/or selected different mutations as a result of their interactions with the microenvironment rather than as an effect of the radiation and temozolomide treatment, although the latter cannot be entirely excluded. It has been shown that disruption of the FA pathway confers genome instability and sensitizes glioma cells to temozolomide [4]. We may speculate that the *FANCD2* germline mutation may have facilitated the relatively good overall response to temozolomide of this patient. However, it is not clear if its LOH in the temporal tumor induced the multiple chromosomal losses that distinguished it from the adjacent frontal tumor (see Fig. 3b).

Glioblastoma is a heterogeneous histologic and genetic entity and efforts are ongoing to subcategorize it based on morphological-molecular correlations [8]. An important pathological finding was that the same four-gene core mutation signature conferred a wide morphologic variation to the tumors, ranging from gliosarcoma to epithelioid glioblastoma. *MET* overexpression in the primary gliosarcoma and the lung metastasis may be responsible for the fibroblast-like morphology of these tumors and possibly, the metastatic potential, as c-MET is a receptor tyrosine kinase involved in epithelial to mesenchymal transition, invasion and metastasis [24]. Both *MET* and *VEGFA* are hypoxia-regulated genes through the activation of HIF-1α [5, 26], and both these tumors were extensively necrotic, most likely mounting a strong anti-hypoxic response. It is not clear how the supratentorial recurrences adopted an epithelial morphology, and unknown environmental cues may have stimulated a reverse cellular mesenchymal to epithelial transition response. Expression of membrane-bound NHERF1 in these tumors but not in the initial gliosarcoma supports this hypothesis, as subcellular transitions of NHERF1 have been linked to the reversibility of these morphological changes [13].

## Conclusions

Although multifocal glioblastoma has been studied and reproduced experimentally in mouse models [17, 20], this is the first temporospatial integrated analysis of multiple tumors arising in a human subject with recurrent, multifocal, multicentric and metastatic glioblastoma. This study brings significant advances to the pathogenesis of metastatic glioblastoma and opens avenues for treatment considerations in these patients.

## Supplementary information

**Additional file 1:** **Table S1.** Tempus xT 596-gene panel. **Table S2.** IHC results. **Table S3.** CN array. **Table S4.** Prevalence of gene mutations in The Cancer Genome Atlas (TCGA,http://www.cbioportal.org/). **Figure S1.** Radiologic progression of recurrent foci. A. Axial T1W pre- and post-contrast images show residual growing enhancing masses within or adjacent to the resection cavity wall, 2 and 3 months after the 2nd resection of both Frontal and Temporal recurrences (arrows). B. Preoperative sagittal T1W pre- and post-contrast images show the rim-enhancing right cerebellar mass. **Figure S2.** Structural mapping of the FANCD2 I273V mutation. Ribbon (upper) and surface 3D (lower) representations of FANCD2 (blue) in complex with FANCI (green). The mutant residue at position 273 is shown in red. Alpha helices are shown as cylinders, and the side chain of the Ile and Val residues as spheres. Note mild reduction in surface hydrophobicity by the Ile to Val change. Protein Data Base of the crystal structure accession number: 3s4w.

## Data Availability

All data generated and analyzed during this study will be deposited in COSMIC repository, https://cancer.sanger.ac.uk/cosmic.

## Abbreviations

CN: Copy number
CNS: Central nervous system
CSF: Cerebrospinal fluid
CT: Computed tomography
EGFR: Epidermal growth factor receptor
FLAIR: Fluid attenuated inversion recovery
GFAP: Glial fibrillary acidic protein
H&E: Hematoxylin eosin
IHC: Immunohistochemistry
LOH: Loss of heterozygosity
MRI: Magnetic resonance imaging
NGS: Next generation sequencing
NHERF1: Na-H exchanger 3 regulatory factor 1
PI3K/*PIK3CA*: Phosphatidylinositol 3-OH kinase catalytic subunit alpha
SNP: Single nucleotide polymorphism
TMB: Tumor mutation burden
VAF: Variant allele fraction
W: Weighted (on MRI sequences T1 and T2)
WHO: World Health Organization
