# Supplementary material for: Genetic and histologic spatiotemporal evolution of recurrent, multifocal, multicentric and metastatic glioblastoma
Source: Acta Neuropathol Commun. 2020 Feb 3;8:10. doi: 10.1186/s40478-020-0889-x (PMC6998196; doi:10.1186/s40478-020-0889-x)
Supplement: Supplementary file 1 — Additional file 1: Table S1. Tempus xT 596-gene panel. Table S2. IHC results. Table S3. CN array. Table S4. Prevalence of gene mutations in The Cancer Genome Atlas (TCGA,http://www.cbioportal.org/). Figure S1. Radiologic progression of recurrent foci. A. Axial T1W pre- and post-contrast images show residual growing enhancing masses within or adjacent to the resection cavity wall, 2 and 3 months after the 2nd resection of both Frontal and Temporal recurrences (arrows). B. Preoperative sagittal T1W pre- and post-contrast images show the rim-enhancing right cerebellar mass. Figure S2. Structural mapping of the FANCD2 I273V mutation. Ribbon (upper) and surface 3D (lower) representations of FANCD2 (blue) in complex with FANCI (green). The mutant residue at position 273 is shown in red. Alpha helices are shown as cylinders, and the side chain of the Ile and Val residues as spheres. Note mild reduction in surface hydrophobicity by the Ile to Val change. Protein Data Base of the crystal structure accession number: 3s4w. [file 40478_2020_889_MOESM1_ESM.pdf]

SUPPLEMENTAL TABLES

Table S1. Tempus xT 596-gene panel.

ABCB1	CFTR	FGF6	INPP4B	NUDT15	SH2B3
ABCC3	CHD2	FGF7	IRF1	NUP98	SLC26A3
ABL1	CHD4	FGF8	IRF2	P2RY8	SLC47A2
ABL2	CHEK1	FGF9	IRF4	PAK1	SLIT2
ACTA2	CHEK2	FGFR1	IRS2	PALB2	SLX4
ACVR1B	CIC	FGFR2	ITPKB	PALLD	SMAD2
AJUBA	CIITA	FGFR3	JAK1	PARK2	SMAD3
AKT1	CKS1B	FGFR4	JAK2	PAX3	SMAD4
AKT2	CREBBP	FH	JAK3	PAX5	SMARCA1
AKT3	CRKL	FHIT	JUN	PAX7	SMARCA4
ALK	CRLF2	FLCN	KAT6A	PAX8	SMARCB1
AMER1	CSF1R	FLT1	KDM5A	PBRM1	SMARCE1
APC	CSF3R	FLT3	KDM5C	PCBP1	SMC1A
APLNR	CTC1	FLT4	KDM6A	PDCD1	SMC3
APOB	CTCF	FNTB	KDR	PDCD1LG2	SMO
AR	CTLA4	FOXA1	KEAP1	PDGFRA	SOCS1
ARAF	CTNNA1	FOXL2	KEL	PDGFRB	SOD2
ARHGAP26	CTNNB1	FOXO1	KIF1B	PDK1	SOX10
ARHGAP35	CTRC	FOXO3	KIT	PDPK1	SOX2
ARID1A	CUX1	FOXP1	KLHL6	PHF6	SOX9
ARID1B	CXCR4	FOXQ1	KLLN	PHOX2B	SPEN
ARID2	CYLD	FRS2	KMT2A	PIAS4	SPINK1
ARID5B	CYP1B1	FUBP1	KMT2B	PIK3C2B	SPOP
ASNS	CYP2D6	G6PD	KMT2C	PIK3CA	SPRED1
ASXL1	CYP3A5	GALNT12	KMT2D	PIK3CB	SRC
ATIC	DAXX	GATA1	KRAS	PIK3CD	SRSF2
ATM	DDB2	GATA2	LAG3	PIK3CG	STAG2
ATP7B	DDR2	GATA3	LDLR	PIK3R1	STAT3
ATR	DDX3X	GATA4	LEF1	PIK3R2	STAT4
ATRX	DICER1	GATA6	LMNA	PIM1	STAT5A
AURKA	DIRC2	GEN1	LMO1	PLCG2	STAT5B
AURKB	DIS3	GLI1	LRP1B	PML	STAT6
AXIN1	DIS3L2	GNA11	LYN	PMS1	STK11
AXIN2	DKC1	GNA13	LZTR1	PMS2	SUFU
AXL	DNM2	GNAQ	MAD2L2	POLD1	SUZ12
B2M	DNMT3A	GNAS	MAF	POLE	SYK
BAP1	DOT1L	GPC3	MAFB	POLH	TAF1
BARD1	DPYD	GPS2	MALT1	POT1	TANC1
BCL10	DYNC2H1	GREM1	MAP2K1	POU2F2	TAP1
BCL11B	EBF1	GRIN2A	MAP2K2	PPARG	TAP2
BCL2	ECT2L	GRM3	MAP2K4	PPP1R15A	TBC1D12
BCL2L1	EGF	GSTP1	MAP3K1	PPP2R1A	TBL1XR1
BCL2L11	EGFR	H19	MAP3K7	PPP2R2A	TBX3
BCL6	EGLN1	H3F3A	MAPK1	PPP6C	TCEB1
BCL7A	ELF3	HAS3	MAX	PRCC	TCF3
BCLAF1	ENG	HAVCR2	MC1R	PRDM1	TCF7L2
BCOR	EP300	HDAC1	MCL1	PREX2	TCL1A
BCORL1	EPCAM	HDAC2	MDM2	PRKAR1A	TERT*
BCR	EPHA2	HDAC4	MDM4	PRSS1	TET2
BIRC3	EPHA7	HGF	MED12	PTCH1	TGFBR2

BLM	EPHB1	HIF1A	MEF2B	PTCH2	TIGIT
BMPR1A	EPHB2	HIST1H1E	MEN1	PTEN	TMEM127
BRAF	EPOR	HIST1H3B	MET	PTPN11	TMEM173
BRCA1	ERBB2	HIST1H4E	MGMT	PTPN13	TMPRSS2
BRCA2	ERBB3	HLA-A	MIB1	PTPN22	TNF
BRD4	ERBB4	HLA-B	MITF	PTPRD	TNFAIP3
BRIP1	ERCC1	HLA-C	MKI67	QKI	TNFRSF14
BTG1	ERCC2	HLA-DMA	MLH1	RAC1	TNFRSF17
BTK	ERCC3	HLA-DMB	MLH3	RAD21	TNFRSF9
BUB1B	ERCC4	HLA-DOA	MLLT3	RAD50	TOP1
C10orf54	ERCC5	HLA-DOB	MPL	RAD51	TOP2A
C11orf30	ERCC6	HLA-DPA1	MRE11A	RAD51B	TP53
C11orf65	ERG	HLA-DPB1	MS4A1	RAD51C	TP63
C3orf70	ERRFI1	HLA-DPB2	MSH2	RAD51D	TPM1
C8orf34	ESR1	HLA-DQA1	MSH3	RAD54L	TPMT
CALR	ETS1	HLA-DQA2	MSH6	RAF1	TRAF3
CARD11	ETS2	HLA-DQB1	MTAP	RANBP2	TSC1
CASP8	ETV1	HLA-DQB2	MTHFR	RARA	TSC2
CASR	ETV4	HLA-DRA	MTOR	RASA1	TSHR
CBFB	ETV5	HLA-DRB1	MTRR	RB1	TUSC3
CBL	ETV6	HLA-DRB5	MUTYH	RBM10	TYMS
CBLB	EWSR1	HLA-DRB6	MYB	RECQL4	U2AF1
CBLC	EZH2	HLA-E	MYC	RET	UBE2T
CBR3	FAM175A	HLA-F	MYCL	RHOA	UGT1A1
CCDC6	FAM46C	HLA-G	MYCN	RICTOR	UGT1A9
CCND1	FANCA	HNF1A	MYD88	RINT1	UMPS
CCND2	FANCB	HNF1B	MYH11	RIT1	VEGFA
CCND3	FANCC	HOXB13	NBN	RNF139	VHL
CCNE1	FANCD2	HRAS	NCOR1	RNF43	WEE1
CD19	FANCE	HSP90AA1	NCOR2	ROS1	WHSC1
CD22	FANCF	HSPH1	NF1	RPL5	WRN
CD274	FANCG	IDH1	NF2	RPS15	WT1
CD40	FANCI	IDH2	NFE2L2	RPS6KB1	XPA
CD70	FANCL	IDO1	NFKBIA	RPTOR	XPC
CD79A	FANCM	IFIT1	NHP2	RSF1	XPO1
CD79B	FAS	IFIT2	NKX2-1	RUNX1	XRCC1
CDC73	FAT1	IFIT3	NOP10	RUNX1T1	XRCC2
CDH1	FBXO11	IFNAR1	NOTCH1	RXRA	XRCC3
CDK12	FBXW7	IFNAR2	NOTCH2	SCG5	YEATS4
CDK4	FCGR2A	IFNGR1	NOTCH3	SDHA	ZFHX3
CDK6	FCGR3A	IFNGR2	NPM1	SDHAF2	ZNF217
CDK8	FDPS	IFNL3	NQO1	SDHB	ZNF471
CDKN1A	FGF1	IKBKE	NRAS	SDHC	ZNF620
CDKN1B	FGF10	IKZF1	NRG1	SDHD	ZNF750
CDKN1C	FGF14	IL10RA	NSD1	SEC23B	ZNRF3
CDKN2A	FGF2	IL15	NT5C2	SEMA3C	ZRSR2
CDKN2B	FGF23	IL2RA	NTHL1	SETBP1	
CDKN2C	FGF3	IL6R	NTRK1	SETD2	
CEBPA	FGF4	IL7R	NTRK2	SF3B1	
CEP57	FGF5	ING1	NTRK3	SGK1	

* Includes promoter region

Table S2. IHC results.

IHC	**Frontotemporal** Gliosarcoma	**Frontal** Ependymoma-tous GBM	**Temporal** Epithelioid[1] GBM	**Cerebellum** GBM	**Lung** GBM
GFAP	+glial<br>-sarcomatous	+	-	+	+
Reticulin[2]	-glial<br>+sarcomatous	NP[3]	NP	NP	+focal[2]
IDH1-R132H	-	-	-	-	NP
Olig2	-	-	+focal	NP	NP
p53[4]	-	-	NP	NP	NP
NHERF1	-	+microlumens	+membranous	NP	NP
Cam 5.2	-	-	+focal	NP	-
PAX8	-	NP	+focal	NP	NP
Synaptophysin	NP	-	-	NP	NP
Ki-67	10%	25.5%	NP	28%	18%

[1]For the epithelioid glioblastoma, the following IHC stains were negative: S100, TTF-1, Napsin A, HBM45, GATA3, Estrogen receptor, Progesterone receptor.

[2]Reticulin, special stain used to identify a sarcomatous component in glioblastoma. The focal nodular deposition in the diffusely GFAP-positive lung tumor may have originated from destroyed alveoli.

[3]NP, not performed.

[4]p53: - when most nuclei negative.

Table S3. CN array.

Start and stop positions are given relative to [GRCh37]

Frontal:

Chr:Start-Stop	Cyto-bands	CN	Comments
chr1:206,654,738-231,296,975	1q32.1 - q42.2	1	60% deletion 1q*
chr3:117,521,471-129,371,519	3q13.32 - q22.1	1	30% deletion 3q
chr5:30,401,682-31,465,137	5p13.3	1	60% deletion 5p
chr5:68,119,704-71,105,662	5q13.1 - q13.2	1	30% deletion 5q
chr7:1-91,598,817	7p22.3 - q21.2	3	60% +7pq
chr7:91,785,970-130,080,812	7q21.2 - q32.2	3	30% +7q
chr7:130,080,812-159,138,663	7q32.2 - q36.3	3	60% +7q
chr10:1-21,616,815	10p15.3 - p12.31	1	60% -10*
chr10:21,616,815-26,778,606	10p12.31 - p12.1	0	independent clone 10% homozygous deletion nested
chr10:26,782,711-135,534,747	10p12.1 - q26.3	1	60% -10* (including *PTEN*)
chr13:19,147,562-46,694,900	13q11 - q14.13	1	60% -13*
chr13:46,694,900-46,788,414	13q14.13	0	60% homozygous deletion nested
chr13:46,788,414-47,335,662	13q14.13 - q14.2	1	60% -13*
chr13:47,335,662-47,826,159	13q14.2	0	60% homozygous deletion nested
chr13:47,826,159-49,032,471	13q14.2	1	60% -13*
chr13:49,032,471-49,367,849	13q14.2	0	60% homozygous deletion nested (including *RB1*)
chr13:49,367,849-115,169,878	13q14.2 – q34	1	60% -13*
chr17:1-20,682,586	17p13.3 – p11.2	2	60% LOH 17p (including *TP53*)
chr19:1-1,390,336	19p13.3	3	60% duplication 19p*
chr19:1,390,336-3,434,224	19p13.3	4	60% high copy gain 19p*
chr19:3,452,637-6,763,718	19p13.3	1	60% deletion 19p*
chr19:6,763,718-8,371,901	19p13.3 – p13.2	4	60% high copy gain 19p*
chr19:8,373,778-19,790,159	19p13.2 – p13.11	3	60% duplication 19p*
chr22:16,197,021-51,304,566	22q11.1 – q13.33	1	60% -22*
chrX:29,845,461-55,518,239	Xp21.2 – p11.21	1	30% deletion Xp

*Same in the Frontal and Temporal tumors.

Temporal:

Chr:Start-Stop	Cyto-bands	CN	Comments*
chr1:99,994,648-206,317,334	1p21.2 - q32.1	1	5% deletion 1pq
chr1:206,614,772-231,273,550	1q32.1 - q42.2	1	25% deletion 1q*
chr1:231,361,110-249,250,621	1q42.2 - q44	1	5% deletion 1q
chr2:1-55,298,517	2p25.3 - p16.1	1	10% deletion 2p
chr2:61,304,909-66,568,270	2p15 - p14	3	10% duplication 2p
chr2:66,568,270-68,298,424	2p14	4	10% high copy gain 2p
chr2:70,196,497-77,681,802	2p13.3 - p12	3	10% duplication 2p
chr2:77,681,802-80,972,177	2p12	4	10% high copy gain 2p
chr2:80,972,177-85,253,059	2p12 - p11.2	3	10% duplication 2p
chr2:175,163,335-243,199,373	2q31.1 - q37.3	0	5% homozygous loss 2q
chr3:1-37,716,880	3p26.3 - p22.2	1	10% deletion 3p
chr3:133,602,133-136,715,468	3q22.1 - q22.3	1	10% deletion 3q
chr4:1-191,154,276	4p16.3 - q35.2	1	5% -4
chr7:1-159,138,663	7p22.3 - q36.3	2	35% LOH 7
chr7:77,582,265-159,138,663	7q21.11 - q36.3	3	35% amplified LOH 7q
chr8:1-89,907,014	8p23.3 - q21.3	1	10% -8pq
chr10:1-135,534,747	10p15.3 - q26.3	1	35% -10* (including *PTEN*)
chr11:1-135,006,516	11p15.5 - q25	1	5% -11
chr12:1-34,337,026	12p13.33 - p11.1	1	5% -12p
chr12:38,359,611-49,245,630	12q12 - q13.12	2	5% 12q LOH

chr12:49,398,863-133,851,895	12q13.12 - q24.33	1	5% large deletion 12q
chr13:19,263,735-115,169,878	13q11 - q34	1	35% -13*
chr14:19,280,733-107,349,540	14q11.2 - q32.33	1	5% -14
chr15:47,438,046-51,285,832	15q21.1 - q21.2	1	5% deletion 15q
chr17:1-12,903,599	17p13.3 - p12	2	25% deletion 17p (including *TP53*)
chr17:12,903,599-20,751,144	17p12 - p11.2	3	25% LOH 17p
chr17:20,766,280-31,509,283	17p11.2 - q11.2	2	25% deletion 17q
chr17:31,509,283-34,991,217	17q11.2 - q12	2	25% duplication 17q
chr17:34,942,595-43,685,925	17q12 - q21.31	3	25% LOH 17q
chr17:43,685,925-52,970,741	17q21.31 - q22	1	25% duplication 17q
chr17:53,018,552-59,543,154	17q22 - q23.2	2	25% LOH 17q
chr17:59,833,137-64,233,289	17q23.2 - q24.2	1	25% LOH 17q
chr17:64,233,289-81,195,210	17q24.2 - q25.3	3	25% duplication 17q
chr18:1-78,077,248	18p11.32 - q23	1	25% -18
chr19:1-1,408,889	19p13.3	3	25% duplication 19p*
chr19:1,408,889-3,452,637	19p13.3	4	25% high copy gain 19p*
chr19:3,481,578-6,722,022	19p13.3	1	25% deletion 19p*
chr19:6,755,155-8,305,642	19p13.3 - p13.2	4	25% high copy gain 19p*
chr19:8,305,642-19,776,814	19p13.2 - p13.11	3	25% duplication 19p*
chr21:14,613,203-19,910,453	21q11.2 - q21.1	1	5% deletion 21q
chr22:16,197,021-51,304,566	22q11.1 - q13.33	1	35% -22*
chrX:1-71,350,133	Xp22.33 - q13.1	1	10% -Xpq

*Same in the Frontal and Temporal tumors.

Table S4. Prevalence of gene mutations in The Cancer Genome Atlas (TCGA,

http://www.cbioportal.org/)[1].

**Mutations**[2,3] (%)	**Glioblastoma** n=1035 samples	**Lung AC**[4] n=1026 samples	**Lung SCC**[4] n=841 samples	**Melanoma** n=808 samples
PTEN	30.0	1.6	9.4	9.8
RB1	8.3	5.3	6.5	4.5
TP53	28.3	49.5	83	15.8
GRIN2A	3.1	10.4	8.7	24.5
ATM	1.5	8.7	5.5	7.5
ZFHX3	1.5	4.5	7.5	12.7
PIK3CA	9.5	5.7	12.8	4.1
PIK3CG	2.5	4.8	8.3	8
SF3B1	0.7	2.5	2.7	5.8
NOTCH1	0.3	4.4	8	6.3
FANCD2	0.9	0.9	0.7	5.3
IDH1	5.3	1.2	0.8	5.7

[1]For the indicated malignancy, all the TCGA-only studies were pooled for analysis.

[2]The genes with mutations in the patient's samples are listed, except for *TERT*, which is not included in the TCGA. *IDH1* mutations (grey shading) are not present in the patient's tumors but are included in the table, as the incidence of *PIK3CA* mutations in IDH wild-type glioblastoma might be overestimated by the presence of a small number of IDH-mutant samples.

[3]The top four mutated genes from this list are highlighted in yellow in each malignancy.

[4]AC, adenocarcinoma; SCC, squamous cell carcinoma.

SUPPLEMENTAL FIGURES

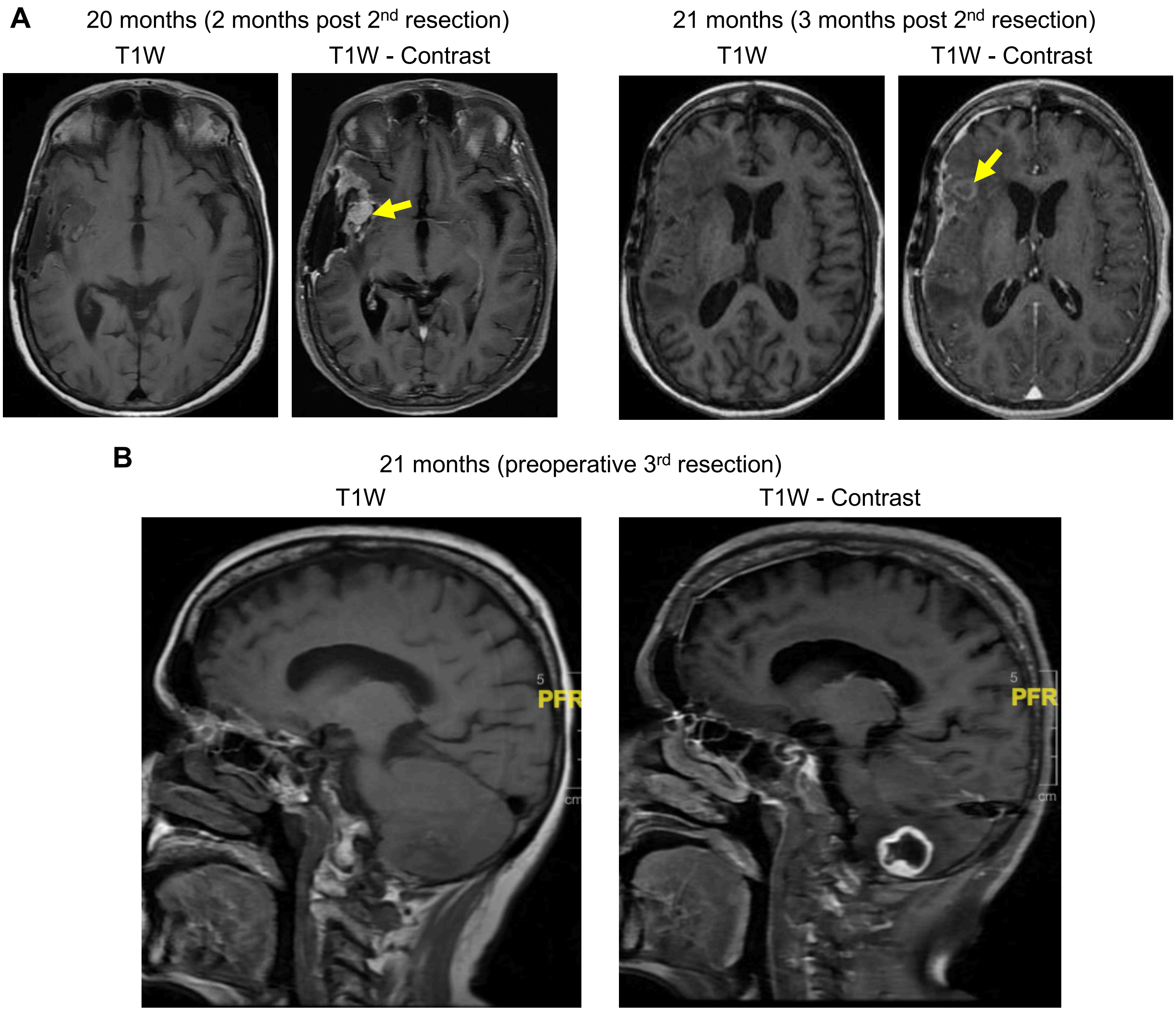

Figure S1. Radiologic progression of recurrent foci. A. Axial T1W pre- and post-contrast images show residual growing enhancing masses within or adjacent to the resection cavity wall, 2 and 3 months after the 2nd resection of both Frontal and Temporal recurrences (arrows). **B.** Preoperative sagittal T1W pre- and post-contrast images show the rim-enhancing right cerebellar mass.

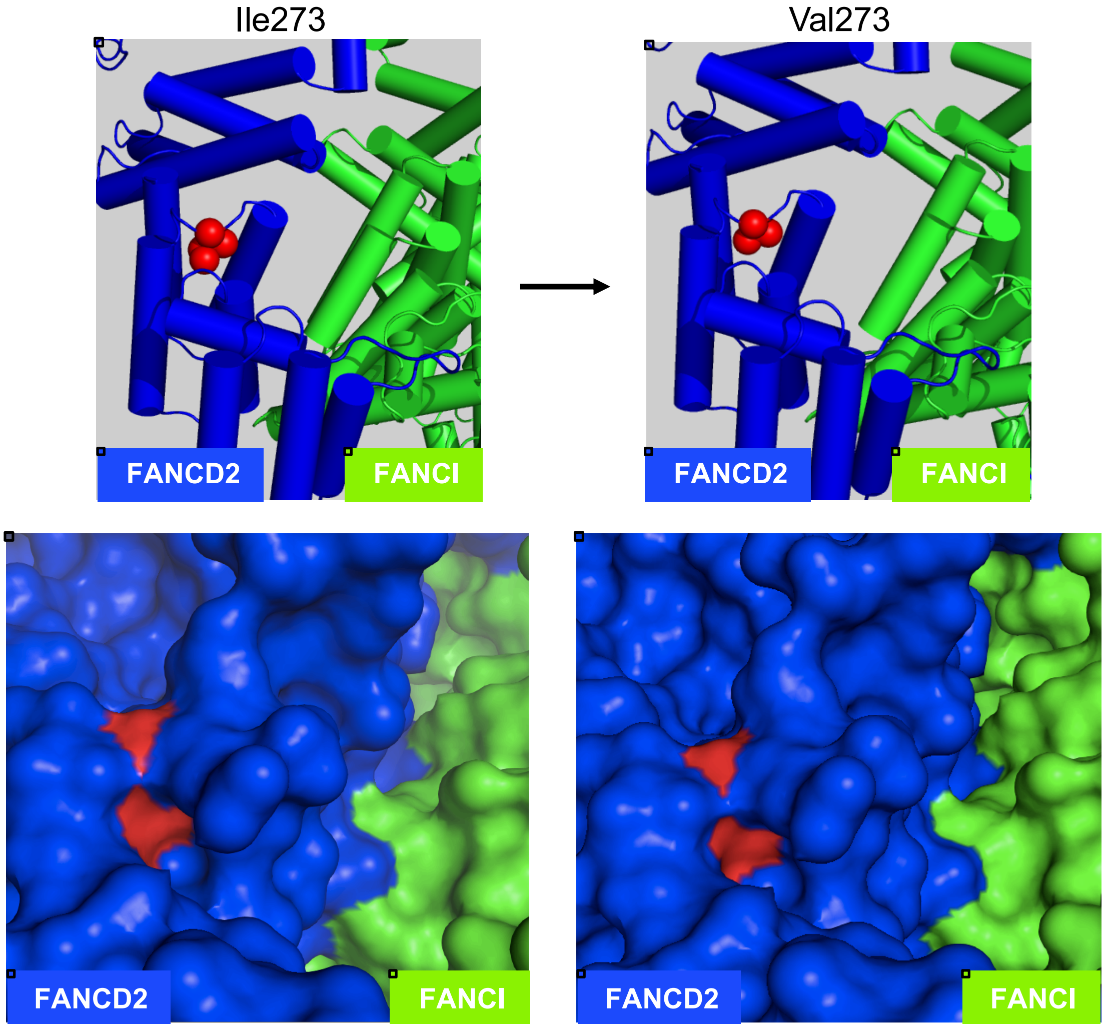

Figure S2. Structural mapping of the FANCD2 I273V mutation. Ribbon (upper) and surface 3D (lower) representations of FANCD2 (blue) in complex with FANCI (green). The mutant residue at position 273 is shown in red. Alpha helices are shown as cylinders, and the side chain of the Ile and Val residues as spheres. Note mild reduction in surface hydrophobicity by the Ile to Val change. Protein Data Base of the crystal structure accession number: 3s4w.
